# Radiotherapy with 16 Gy may fail to eradicate testicular intraepithelial neoplasia: preliminary communication of a dose-reduction trial of the German Testicular Cancer Study Group

**DOI:** 10.1038/sj.bjc.6600771

**Published:** 2003-03-18

**Authors:** J Classen, K Dieckmann, M Bamberg, R Souchon, S Kliesch, M Kuehn, V Loy

**Affiliations:** 1 Department of Radiation Oncology, Tuebingen University, Hoppe-Seyler-Strasse. 3, D-72074 Tuebingen, Germany; 2 Department of Urology, Albertinen-Krankenhaus, Suentelstrasse 11a, D-22457 Hamburg, Berlin, Germany; 3 Department of Radiation Oncology, Allgemeines Krankenhaus, Gruenstrasse 35, D-58095 Hagen, Berlin, Germany; 4 Department of Urology, Muenster University, Albert-Schweitzer-Strasse 33, D-48129 Muenster, Germany; 5 Department of Urology, Johanniter-Krankenhaus, Wendstraße 31, D-39576 Stendal, Germany; 6 Department of Pathology, Krankenhaus am Urban, Dieffenbachstrasse 1, D-10967 Berlin, Germany

**Keywords:** testicular intraepithelial neoplasia, testicular neoplasms, radiotherapy, spermatogonia, testicular biopsy, bilateral testicular neoplasms

## Abstract

Low-dose radiotherapy to the testis is effective in eradicating testicular intraepithelial neoplasia (TIN, carcinoma *in situ* of the testis) at the risk of androgenic deficiency. The present trial was designed to define the lowest dose effective to control TIN assuming a dose–response relation of radiation-induced endocrinological damage. Patients with TIN in a solitary testicle or with bilateral TIN were treated with 18 Gy (14 patients) and 16 Gy (26 patients) (5 × 2 Gy per week). Biopsies to ascertain clearance of TIN were performed after 6 and 24 months. The median time of follow-up is 20.5 months. There were three adverse events. In one patient, relapse of TIN along with microinvasive seminoma was observed 2 years after 16 Gy irradiation. In two other patients, persistent spermatogonia were observed with the 16 and 18 Gy regimen after 6 and 24 months, respectively. All other post-treatment biopsies showed the Sertoli cell-only pattern. These results confirm that TIN is a radiosensitive lesion efficiently controlled in most cases with doses below 20 Gy. However, sporadic failures may occur. A dose of 16 Gy is probably unsafe and should no longer be used. Future investigations should not only focus on total dosage of irradiation but also on fractionation schedules.

## 

Testicular intraepithelial neoplasia (TIN; also called carcinoma *in situ* of the testis) is the uniform precursor of testicular germ cell tumours (Dieckmann and Skakkebaek, 1999). According to the current theory of the histogenesis of testicular tumours, TIN evolves from embryonic germ cells and thus it is present in the testicle many years before the tumour becomes invasive. Morphologically, TIN consists of large intratubular cells that closely resemble embryonic gonocytes (Rorth *et al*, 2000). Diagnosis is achieved by testicular biopsy and immunohistological examination with staining for placental alkaline phosphatase (PlAP). If TIN is left untreated after diagnosis, malignancy will develop in 50% of cases after 5 years and in 70% after 7 years, respectively. Probably, all cases of TIN will ultimately proceed to invasive testicular cancer (Skakkebaek *et al*, 1987). TIN can be effectively treated by low-dose radiotherapy. This treatment is appealing because TIN and thus the risk of testis cancer is eliminated without surgical castration. Moreover, the testosterone-producing Leydig cells are largely preserved and, consequently, hormone supplementation is usually not required in these individuals. So far, experience with radiotherapy of TIN is limited. According to early experience (von der Maase *et al*, 1986; Giwercman *et al*, 1991; Dieckmann *et al*, 1993), a total dose of 18–20 Gy is sufficient to clear the testis from TIN (Bamberg *et al*, 1997); however, it became obvious that this dosage also causes damage to the Leydig cells in at least one quarter of the patients (Dieckmann *et al*, 2001; Classen *et al*, 2001), rendering these patients dependent on androgen substitution. Therefore, a clinical trial was initiated to look for the most appropriate radiation dose that is still sufficient to eradicate TIN effectively, and that preserves Leydig cell function at the same time. Owing to unexpected outcomes, we here report interim results of this trial with respect to control of TIN by radiotherapy. The endocrinological data are still premature and will be reported later.

### Patients and methods

A two-stage phase II study design was used for the trial (Simon, 1989). A stepwise dose-reduction schedule was employed starting at the 18 Gy level with dose-reduction steps of 2 Gy each. Dosage decrease was scheduled until treatment failure at any dose level was experienced or until the 12 Gy level was reached. According to the two-stage design of the trial, dose reduction on each dose level was employed after a minimum of seven patients had completed treatment on this level without adverse events, that is without persistence of TIN or germ cells, respectively, as evidenced by post-treatment biopsy (Simon, 1989). In the case of treatment failure at a given dose level, it was intended to confirm the safety of the corresponding next higher dosage level (+2 Gy) by a further sample of 24 patients for a population of 31 patients on this dose level (Simon, 1989).

Irradiation was applied according to international standards in 2 Gy fractions with five applications per week. The target volume of radiotherapy was prescribed to a mid-testicular reference point, which was set to 100%. The minimum target volume dose allowed was 85% and the maximum volume dose was limited to no more than 107%. Radiotherapy was delivered by a single anterior electron beam field on a linear accelerator with a median energy of 11 MeV (range 4–18 MeV).

Biopsies to ascertain clearance of TIN were performed after 6 months and again after 2 years. In all cases, the histological diagnosis of TIN as well as the absence or persistence of TIN in post-treatment biopsies was confirmed by a reference pathologist (VL). Histological work-up involved conventional histology and immunohistological examination as detailed previously (Loy *et al*, 1990). Treatment failure was defined as the persistence or relapse of TIN as observed histologically by biopsy, and, alternatively, as the occurrence of an invasive testicular tumour as observed clinically. Endocrinological evaluations were done prior to radiotherapy and repeatedly during follow-up. These data will be reported in a separate communication.

After initiation of the trial on the 18 Gy dose level, dose reduction was terminated prematurely at the 16 Gy level because we became aware of a TIN relapse on the 14 Gy level from a concurrent Danish trial (Skakkebaek, personal communication, 1999). According to a protocol amendment, we then proceeded to apply 16 Gy in a patient sample large enough (calculated sample size according to Simon (1989) *n*=31) to prove the safety of this dosage to eradicate TIN.

Patients with histologically proven TIN in a solitary testis or with bilateral TIN and without concurrent chemotherapy were eligible for the trial. Patients had to sign informed consent according to the declaration of Helsinki. The study was approved by an ethical committee. The study was initiated in August 1998 and the present report is based on the cumulative data available on 31 December 2001.

## RESULTS

Forty-three patients were entered on to the trial from 29 participating institutions (appendix). Three patients were excluded because reference histology disclosed invasive seminoma in one patient and the absence of TIN in two others. Thus, 40 patients were evaluable for the study. Five patients had undergone chemotherapy prior to radiotherapy: two had single agent carboplatin (two courses) and three had full chemotherapy courses with cisplatinum, etoposide and bleomycin (PEB). In each of these patients, the persistence of TIN after chemotherapy had been confirmed by biopsy.

Thirty-five patients had TIN in a solitary testicle, all of whom had undergone surgery for a germ cell cancer in their contralateral testicle. The right testis was involved in 19 cases, the left in 12; laterality was unspecified in four patients. Four patients had bilateral TIN. Median age was 30 years (range 20–45 years). Median time of follow-up was 20.5 months (range 0.5–40 months). Fourteen patients received 18 Gy, and 26 underwent the 16 Gy regimen.

Acute toxicity of radiotherapy was low. Six patients experienced skin toxicity grade I (RTOG score), three of whom had received the 16 and 18 Gy regimen, respectively. There was no toxicity >grade I.

**Figure 1:**
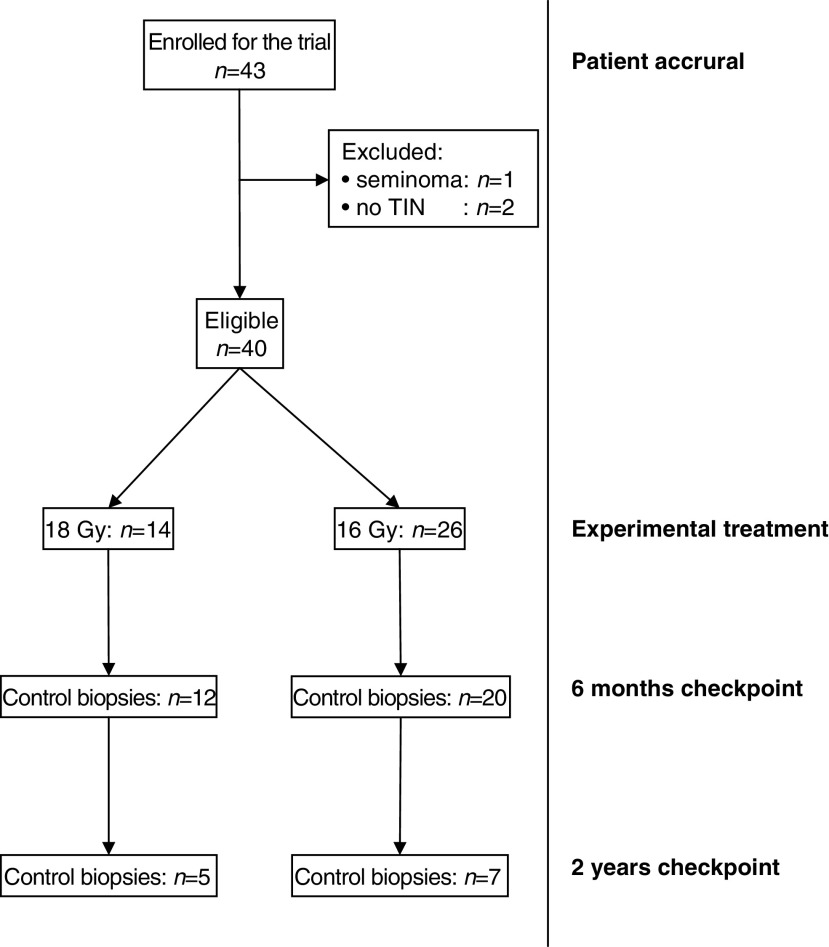
Study population and follow-up biopsies after radiotherapy.

A total of 44 post-treatment biopsies were analysed histologically. The first and second biopsies were taken with a median time to follow-up of 6.2 months (range 2.6–9.0 months) and 25.4 months (range 18.9–28.2 months), respectively. Further statistical details are given in Figure 1

. Histologically, three adverse events were encountered: one relapse of TIN, one patient with persistence of germ cells, and one other with reoccurrence of germ cells.

Overall, no persistence of TIN was observed in any post-treatment biopsy at the 6-month checkpoint. However, in one patient who had undergone the 16 Gy regimen, several vital spermatogonia were observed histologically. A second biopsy taken from this patient after 2 years was clear of TIN and germ cells. Currently, this patient is well and without clinical signs of malignancy.

**Figure 2:**
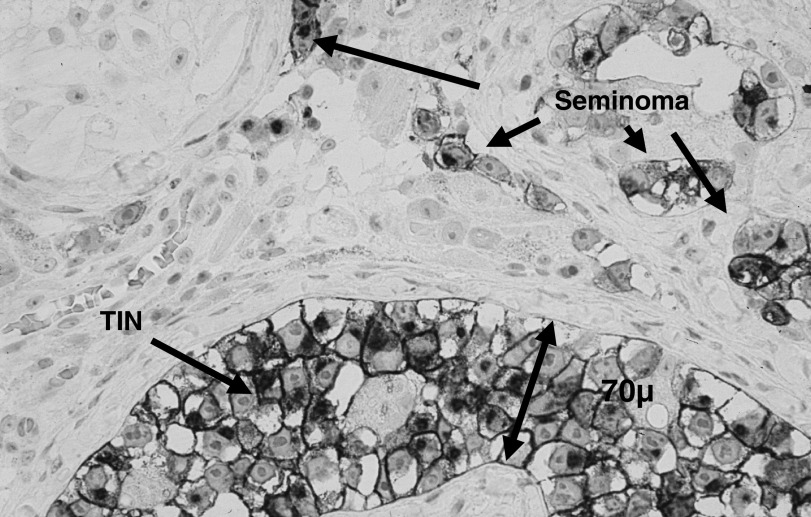
Seminoma and testicular intraepithelial neoplasia after 16 Gy radiotherapy; PlAP, orig. × 120.

At the 2-year checkpoint, 10 biopsies were clear of TIN and germ cells. However, TIN was detected in one patient who had been found free of TIN 6 months after completion of irradiation with 16 Gy. This patient subsequently underwent orchiectomy. Histological evaluation confirmed the presence of TIN in numerous seminiferous tubules and, importantly, it also disclosed the presence of microinvasive seminoma (Figure 2

). Germ cells were absent in the orchiectomy specimen. In a second patient treated with 18 Gy, Sertoli cells only were found 6 months after completion of therapy, but isolated germ cells were detected in the second biopsy 2 years after radiotherapy. This patient is under continuous observation without clinical signs of testicular malignancy.

## DISCUSSION

The finding of a TIN relapse after 16 Gy radiotherapy is clearly nonanticipated. At the outset of the present study, the expectation based on the cumulative international experience (Giwercman *et al*, 1991; Mumperow *et al*, 1992; Dieckmann *et al*, 1993; Classen *et al*, 1998; Kazem and Danella, 1999; Sedlmayer *et al*, 2001) had been that 14 Gy or possibly even 12 Gy radiotherapy would still be sufficient to eradicate TIN.

Failure of chemotherapy to eliminate TIN and likewise its inefficacy of preventing contralateral germ cell tumours has been observed in abundance (Hoff-Wanderas *et al*, 1997; Christensen *et al*, 1998; Ostau *et al*, 2001). Accordingly, there were five patients with persistent TIN after chemotherapy in the present series. Primary resistance of TIN cells to chemotherapy, and possibly, protection from chemotherapy by the blood–testis barrier are the probable biological reasons for the inefficacy of chemotherapy to cure TIN.

The reasons for the relapse of TIN despite radiotherapy are unclear. Technical failure is improbable since in the present case the first post-treatment biopsy had demonstrated the absence of TIN and germ cells.

A feasible explanation would be the hypothesis that some of the TIN cells in the present patient were somehow radioresistant and thus escaped eradication. Presumably, in the present patient a few TIN cells had been present even at the 6-month checkpoint. Obviously, because of their low number and focal arrangement, they escaped detection by biopsy at that time. The surviving TIN cells resumed replication in the later course and were then large enough in number and topographic distribution to be detected by the biopsy at 2 years.

Further, the observation of persisting vital spermatogonia after 16 Gy and even after 18 Gy is incompletely understood, too. Such a finding has not been reported previously. Since spermatogonia and TIN cells share a number of biological features, it must be suspected that the persistence of germ cells after radiotherapy might herald also the persistence of TIN cells.

A further note is that the median follow-up is still low (20.5 months) in the present series. As the relapse has been found in a biopsy after 2 years and only 12 patients have passed this checkpoint to date, it could be suspected that even more relapses might be disclosed during longer follow-up. Thus, the adverse events reported here, although constituting only early experience, clearly indicate that the 16 Gy regimen involves a significant potential of treatment failure, and even the 18 Gy dose level may finally prove to be insufficient for safe eradication of TIN.

Recently, Petersen *et al* (2002) reported the recurrence of TIN after 14 Gy irradiation with a standard fractionation schedule. In analogy to our patient, that relapse was observed 2 years after completion of treatment. In contrast to our study, Petersen *et al* did not encounter relapse of TIN at the 16 Gy level. Furthermore, they did not observe persisting germ cells after the 16 and 18 Gy irradiation schedule. Possibly, small patient samples in both of the studies (the present one and the Petersen study) account for these incongruent findings. Interestingly, endocrinological compromise was present in many of the patients in the Petersen study with no significant differences between the various dose levels. In all, that report strongly accords with our observation that a dose reduction below 18 Gy involves a small but definite risk of treatment failure. In addition, it appears equivocal so far that a dose reduction of radiotherapy will ultimately translate into a substantial clinical benefit for the patient, that is, improved preservation of androgen synthesis.

Clearly, another important lesson to be learned from the present trial is that biopsies to control radiotherapeutic success are paramount. Moreover, late biopsies (e.g. after 2 years) are much more appropriate than early biopsies (i.e. after 6 months). Possibly, even very late biopsies after 3 or 4 years could be useful. If one assumes a persistence of TIN subsequent to radiotherapy, then this condition probably consists of a tiny focus, morphologically. A random biopsy taken 6 months thereafter probably has a large potential to miss that lesion. However, as TIN will inevitably resume replication, biopsies taken during later follow-up have a much higher chance of detecting the condition.

The dose–response relation of TIN is unknown so far. Owing to morphological similarity, it is assumed that TIN and spermatogonia are at least partly comparable with respect to radiosensitivity. Germ cells are highly vulnerable to radiotherapy. Even scatter doses from radiotherapy to abdominal or pelvic target organs can cause significant damage to the germinative epithelium (Classen and Bamberg, 1999). Depletion of germ cells is usually achieved after total doses exceed 12–14 Gy depending on the fractionation schedule (Shalet, 1993).

In contrast to other tissues, germ cells are particularly sensitive to fractionated irradiation. This phenomenon is because of different radiosensitivity of the various stages of germ cell maturation. Type A spermatogonia, the presumed stem cells of spermatogenesis, are rather radioresistant possibly due to their long cell cycle. Type B spermatogonia have a much shorter cycle time, which may be the reason for their increased radiosensitivity.

Based on the morphological and biological similarities of spermatogonia and TIN cells, it could thus be speculated that not only the total dose of radiation but also the fractionation schedule is critical for cure of TIN by radiotherapy. Accordingly, Sedlmayer *et al* (2001) reported the efficacy of a 13 Gy total dose applied in 10 fractions to eradicate TIN at least in a short time to follow-up and in a small cohort of patients.

**Figure 3:**
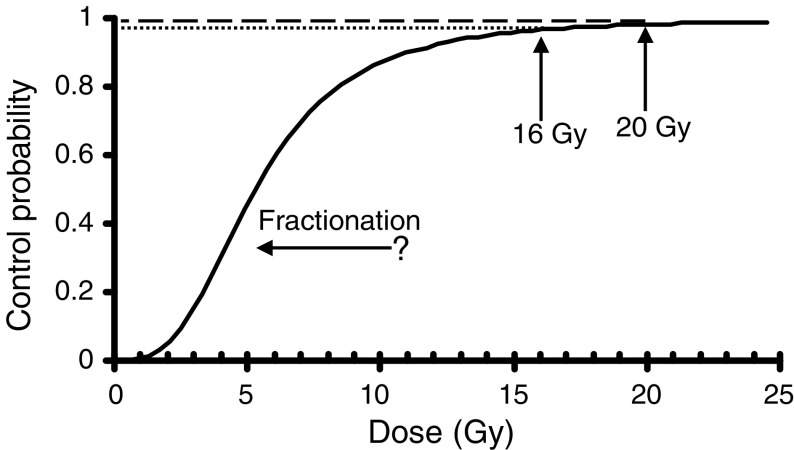
Dose–response curve hypothesised for radiotherapy of TIN. Increasing the number of fractions may shift the response curve to the left.

According to standard fractionation regimens, a dose–response curve as shown in Figure 3

may be hypothesised for local radiotherapy of the testis (Figure 3). Total doses of 16 Gy or more will cure TIN in the majority of cases, but some of the cases will relapse or persist as demonstrated in the present study. Doses of 18–20 Gy will cure TiN in almost 100% of cases. However, sporadic relapse may occur even after standard dose treatment (Dötsch *et al*, 2000; Dieckmann *et al*, 2002). If higher doses are applied, no new growths have been observed (Read, 1987). With regard to the dose–response curve (Figure 3), it may be speculated that the curve could be shifted to the left by decreasing the daily dosage below the classical 2 Gy standard dose and by increasing the number of fractions at the same time. Thus, higher cure rates might be achieved with lower total doses. Furthermore, reduced single doses of treatment might contribute to protect androgen-producing Leydig cells from late sequelae of irradiation.

In conclusion, it becomes obvious that control of TIN by current radiotherapeutic strategies is not possible in virtually 100% of cases. High cure rates are achievable even with doses of radiotherapy just below or around the 20 Gy level, but sporadically, relapses may occur. The optimal dose of radiotherapy is yet to be found. Total doses of 16 Gy with standard fractionation is obviously not safe enough and should no longer be used. Conceivably, a schedule with higher fractionation may offer another convenient avenue to dose reduction and thus preservation of hormone-active Leydig cells.

## Appendix : List of participating institutions

Department of Radiation Oncology (XRT), University Tuebingen, XRT, Klinikum Rechts der Isar Muenchen, Department of Urology (URO), Krankenhaus Luedenscheid, URO, Johanniter Krankenhaus Stendal, URO, University Muenster, URO, University Benjamin Franklin, Berlin, XRT, University Hannover, URO practice Dr Hauschild Hamburg; XRT, Klinikum Offenburg, XRT, Krupp-Krankenhaus Essen, URO, Klinikum Mannheim, URO, Klinikum Am Urban Berlin, XRT, Klinikum Neubrandenburg, XRT, Allgemenines Krankenhaus Hagen, URO, Universität Greifswald, URO, practice Dr Kaup Kiel, XRT, University Goettingen, XRT Klinikum Aschaffenburg, XRT, Dreifaltigkeitshospital Lippstadt, XRT, Klinikum Grosshadern Muenchen, XRT, University Duesseldorf, URO, Klinikum Kemperhof Koblenz, XRT, Klinikum Kassel, URO, practice Dr Warnack Hagenow, URO, Klinikum Wuppertal, XRT, Kreiskrankenhaus Guetersloh, URO, Albertinen-Krankenhaus Hamburg, XRT, Klinikum Chemnitz, XRT, Charité Berlin.
